# Low-Income Population Sugar (Sucrose) Intake: A Cross-Sectional Study among Adults Assisted by a Brazilian Food Assistance Program

**DOI:** 10.3390/nu11040798

**Published:** 2019-04-08

**Authors:** Raquel Braz Assunção Botelho, Rita de Cássia Akutsu, Renata Puppin Zandonadi

**Affiliations:** Department of Nutrition, Faculty of Health Sciences, University of Brasilia, Brasilia 70910-900, Brazil; rita.akutsu@gmail.com (R.d.C.A.); renatapz@yahoo.com.br (R.P.Z.)

**Keywords:** sucrose intake, Brazilian low-income people, popular restaurants

## Abstract

Non-communicable diseases are increasing worldwide, and it has been known that sugar intake is associated with health implications. Studies show that sugar consumption is high among the low-income population. In Brazil, there is a Food Assistance Program to offer inexpensive and healthy meals to the low-income population, aiming to improve their health. However, no study has evaluated either the amount of sugar consumption by the Brazilian low-income population or its distribution among the consumed products. This work aimed to analyze the sugar (sucrose) consumption by the Brazilian low-income population. We carried out a cross-sectional and descriptive study to evaluate the typical customers of a popular restaurant (PR) in Brazil (a Brazilian Food Assistance Program for low-income people). In the final sample, 1232 adult PR customers were surveyed. The exclusion criteria were pregnant women, diabetics, or people following any special diet with sucrose restrictions. Individuals were selected at lunchtime while they were in line waiting to collect their meal. Invitations to participate occurred to the first person in line, then the 15th person, and this pattern was used until the sample was completed. Three-day 24 h recall was used to evaluate sugar consumption. Sociodemographic and anthropometric data were collected to allow profiling of the customers. A statistical analysis of the data with descriptive nature (frequency, mean, median, percentage, and standard deviation) was performed to characterize the sample. For all the analyses, statistical normality tests were performed (Kolmogorov–Smirnov) to verify the statistical test assumptions. The mean total energy value (TEV) over the evaluated three-day period was 1980.23 ± 726.75 kcal. A statistically significant difference was found between income groups (*p* < 0.01). The North and Northeast region presented the lowest mean income in Brazil, statistically different from the South (*p* < 0.01) and the Southeast (*p* < 0.01). The North region presented the lowest sugar intake from industrialized products—different from the Northeast (*p* = 0.007), the Southeast (*p* = 0.010), and the South (*p* = 0.043). Also, the North presented the lowest consumption for food prepared at home among other regions (*p* < 0.001). Total sugar (sucrose) intake did not differ according to body mass index (*p* = 0.321). There was no significant difference in sugar (sucrose) consumption among the three days (*p* = 0.078). The addition of sugar (sucrose) contributed to 36.7% of all sugar (sucrose), and sweetened beverages with 22.53%. Food prepared at home contributed 20.06% and industrialized products 22.53% of the sugar (sucrose) intake. Therefore, free sugar (sucrose) consumption is still the largest contributor to the total consumption of sugar (sucrose), followed by sweetened drinks, especially during the weekend. The average percentage of sugar (sucrose) intake is above the World Health Organization recommendation to consume less than 5% of the total energy that comes from sugars. Since this population presents a high percentage of overweight and obese, the sugar (sucrose) consumption could increase health implications, increasing the costs for public health.

## 1. Introduction

There is a consensus in public health that sugar (sucrose) needs to be reduced from desserts, snacks, and sugar (sucrose)-sweetened beverages [1]. Sugar-sweetened beverages represent the primary source of energy from beverages consumed among North Americans, contributing with 9.2% of the total energy intake [2]. In Brazil, sugar (sucrose) consumption corresponds to 12.9% of the total energetic value of the diet, according to the Brazilian Household Budget Survey (*Pesquisa de orçamento familiar—POF*) [3].

Non-communicable diseases are increasing worldwide, and the association between sugar intake and health implications is known [4,5,6]. A strong body of evidence shows that the putative main effect of sugar is that it provides additional energy [7,8]. A systematic review showed a positive association between a higher intake of sugar (sucrose) and body fat in adults, increasing the prevalence of chronic diseases [9]. In this context, the World Health Organization (WHO) [10] stated in its guidelines, in 2003, that no more than 10% of an adult’s daily energy should come from ‘free’ sugars [11]. Nowadays, the WHO and the Scientific Advisory Committee on Nutrition (SACN) propose to cut the level to 5%, which is equivalent to about 25 g of sugar (sucrose) in a 2000 kcal diet [2,8,12]. In 2018, the Brazilian Department of Health and the food industry sector signed an agreement to reduce sugar consumption in Brazil with the goal of reducing 144 thousand tons of sugar by 2022 [13]. Such agreement follows the trends of public policies to reduce sugar consumption worldwide, such as those practiced in the United Kingdom [14].

Some studies show that sugar consumption is high among the low-income population in some countries [15,16,17]. In Brazil, the government created popular restaurants (PRs) as a Food Assistance Program to offer inexpensive and healthy meals to the low-income population [18,19]. The purpose of this program is to guarantee the social rights of feeding [20] and to improve the health of the low-income population, since food access has a substantial impact on the prevention and treatment of several diseases. In accordance with PR objectives, the government needs to provide a healthy lunch option for those who eat out on a low budget, charging from US$0.30 to US$0.60 per person for each lunch meal. The PR menus are composed of a main course (offered on the menu as a protein preparation, usually of animal origin and decisive for the selection of other items), garnish (a menu item accompanying the main course, which may have vegetables, pasta, tubers, and cassava flour as its main ingredients), side dishes (items such as rice and beans, traditionally consumed by the Brazilian population on a daily basis), salad, and dessert (fruit) [18,21].

The cultivation of sugar cane in Brazil contributes to its wide availability and low cost, strongly influencing its use as an ingredient in several bakery and confectionary products (cake, cookies, bread) in addition to sugar-sweetened beverages. It is hypothesized that there is high sugar consumption among low-income Brazilian populations through the consumption of different types of products. However, no study has evaluated either the amount of sugar consumption by low-income populations or its distribution among consumed products. Studies usually focus on evaluating soft drink consumption and not the pattern of sugar intake. In this context, this study aimed to analyze the consumption of sugar (sucrose) in low-income populations of Brazil.

## 2. Materials and Methods

### 2.1. Design

This cross-sectional study is a result of the cooperation agreement signed between the Department for Social Development and Hunger Fighting (*Ministério do Desenvolvimento Social e Combate à Fome*, MDS) [19] with the University of Brasilia. The sample calculation was performed based on the official list of popular restaurants (PRs) from the program of this department. These popular restaurants are part of the Brazilian Food Assistance Program. In order to select PRs, the following inclusion criteria were used: (i) a food service belonging to the Popular Restaurants program of the Brazilian Federal Government; (ii) signature of the Institutional Acknowledgment Agreement by the personresponsible for the food service; (iii) lunch opening hours; and (iv) service of more than 500 meals daily. Given the inclusion criteria, all of the 65 existing PRs were eligible to be part of the study. From the PRs (N), the sampling plan was calculated considering an error (e) of a daily meal and a level of significance (α) of 5% [22]. A simple random sample was estimated through the procedure “survey select” of the SAS 9.1.3 program. From the 65 PRs and the sampling plan, a minimum of 32 PRs should be visited to complete the study. From the existing PRs in each region, units were drawn by researchers to be part of the sample, respecting the stratification criteria for each of the five Brazilian geographical regions.

Each PR was required to have a minimum sample of 37 customers. The PRs included in this study were located in urban centers where there is considerable human traffic, which facilitates people´s access to the PRs [19]. The estimate of individuals per PR was made using G-Power 3.0.10 software, which uses statistical test parameters along with the power of the experiment. The inclusion criteria were to be a frequent customer (more than three times a week, according to their response during the interview) and over 18 years old. Excluded from the sample were pregnant women, due to their different nutritional needs; individuals under 18 years; and people who presented with diabetes or followed any particular diet with sucrose restrictions. The selection of individuals occurred while they were waiting in line to collect their meal at lunchtime (self-service). Invitations to participate occurred to the first person in line, then the 15th person, and this pattern was used until the sample was complete. For sampling purposes, users were addressed systematically, that is, 1 out of every 15 who entered the restaurant on the data collection day. They were replaced by the subsequent user when they refused to participate or did not fulfil the assiduity requirement, followed by the 30th, and the 45th, consecutively. In each PR, more than 37 customers were invited to participate in the study, in case people had to be excluded from the study because they did not participate in the three days required for the survey.

On the first day (Monday), participants were recruited, and they signed the acknowledgment and agreement terms, and the evaluation occurred for three days at the PRs. While in line waiting to collect their food, each participant received information about the research, the need to have lunch at the PR for at least two more days, the advice to introduce themselves on the consecutive days of the study, and the procedures for weight and height measurements on Tuesday. Participants were not excluded if they were illiterate, but they needed to sign the acknowledgment and agreement terms which was read by the researcher. After the recruitment, the same participant continued the evaluation for the rest of the study. For this study, participants had to complete the three days of consumption evaluation to qualify. The same individual was interviewed at the restaurant to complete the three-day 24 h recall, in which they were asked about their relevant activities during the whole of the previous day. According to the Institute of Medicine, Food, and Nutrition Board, the evaluation of at least three consecutive days of food consumption is representative of an individual’s diet [23]. For each participant, the study presented one 24 h recall for the weekend, and two 24 h recall for the weekdays. The same researchers conducted interviews with consumers and following a protocol designed for the study.

The evaluation of sociodemographic data was developed by a structured questionnaire with standard questions by a trained researcher. The evaluated variables were gender, age, level of education, and income. The anthropometric measurements of weight and height were taken on the second day of the study in each PR before participants had their lunch and, from this data, the body mass index (BMI) was calculated. The questionnaire also contained questions regarding diet-related diseases and non-communicable diseases.

**Figure 1:**
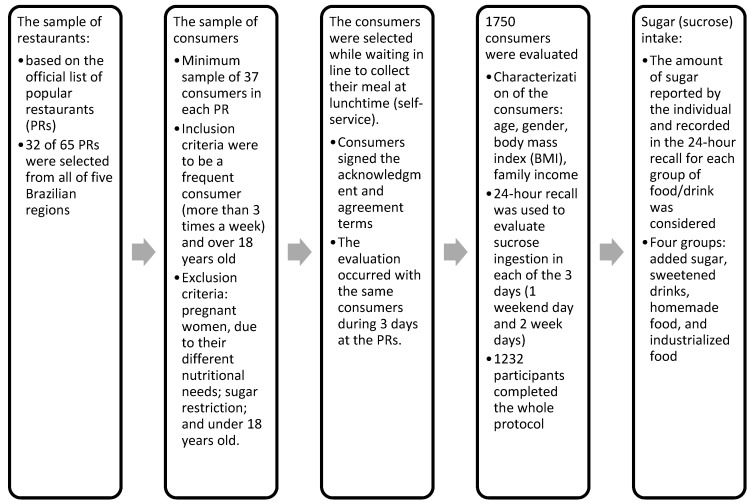
Study design and data collection.

The customers that had not presented 24 h recall data for all of the three days were excluded from the sample. From the 1750 recruited participants, a total of 1232 people that had been randomly and systematically assessed completed the whole protocol. The sampling error for national representativeness was less than 3% (the total number of customers of the PR program in the country was 55,350 individuals/day). It is important to mention that PRs only offer lunch to Brazilian low-income people on weekdays. The summary of the study stages is displayed in Figure 1.

### 2.2. Variables

The customers were interviewed on consecutive three days, and in each day, 24 h recall was applied. On Monday, customers were asked about their consumption at each meal held on Sunday, to characterize the weekend consumption, and the other two days (Tuesday and Wednesday) regarded the weekday consumption. For this analysis, a 24 h recall [24] was used, in which customers described food consumed in the PR and out of PR and their amounts. Researchers could use, if necessary for better accuracy of data collection, utensils and food portion photos.

The only meal participants consumed at the PR was lunch composed of a meat source, rice, beans, salads, and one side dish. Fruits as desserts were not served in all the PRs, and sweet desserts were not offered in any of them. Based on the recorded data, the nutritional values were calculated using the information available in the Brazilian Food Composition Table [25]. When this information did not exist, scientific publications and labels on processed food products were used and then the information was entered into the system database (DietWin^®^, Porto Alegre, Brazil).

On Monday (the first day), sociodemographic information was also collected, such as gender, age, family income, as well as the anthropometric variables (weight, height) of the individual [26]. All data were registered by the researchers. The body mass index (BMI) was used as an indicator of an anthropometric component of nutritional status. Classification occurred according to the criteria adopted by the World Health Organization [27].

In the assessment of the nutritional composition of meals, the total energy value (TEV) of the day of each participant was analyzed, along with their consumption of sugar (sucrose) in each preparation at each evaluation date. For the sugar (sucrose) consumption analysis of each participant, all 24 h recalls were evaluated to identify four consumption groups: (1) added sugar; (2) sweetened drinks; (3) industrialized foods; and (4) sweet food prepared at home.

For added sugar (sucrose), all sugar (sucrose) used by the participant to sweeten coffee, tea, milk, smoothie, porridge, and juice (natural or concentrated without sugar (sucrose)) were measured. The amount of sugar (sucrose) reported by the individual and recorded in the 24 h recall for each type of liquid was considered. For the sucrose from deserts prepared at home (like cakes, puddings, mousses), the typical amount of sucrose used for these recipes in Brazil was considered.

The third assessed item was the consumption of sucrose present in industrialized products, such as sweet biscuits, jellies, sweet pastries, sweet tablets, chocolate, candies, ice cream, popsicles, breakfast cereal, granola, condensed milk, sweetened yoghurt, ketchup, and cereal bars, among others. For sucrose in each of these products, label information, product websites, and data provided by Proteste [28] were used. The fourth assessed item was the amount of sucrose within soft drinks of any flavor, juices, ready to drink nectars, powdered drinks with sucrose, and soy drinks with fruit juice, and were evaluated similarly to the procedure cited for the third item.

Thus, for each of the three days, four groups of sugar (sucrose) were evaluated. The percentage of energy provided was estimated by these four groups concerning the consumption of TEV of each participant to allow for comparison with WHO recommendations, i.e., up to 10% of the energy intake from consumption of sucrose and preferably below 5% [11].

### 2.3. Statistical Analysis

Nutritional information was corrected by variability among people and within each person using MSM software to estimate the usual intake of nutrients by the population. The statistical analysis of this study was processed by SPSS 24.0^®^ software, using descriptive variables, correlations, parametric tests, and the *t*-test and Tukey test for ANOVA to verify statistical differences in the data between regions. It was established that *p* < 0.05 was statistically significant. Outliers (participants with disparate high sugar intake) were removed after analyzing data.

The Statistical Package Program for Science—SPSS, version 24.0, was used in statistical analyses. A statistical analysis of the data with descriptive nature (frequency, mean, median, percentage, and standard deviation) was performed to characterize the sample. For all the analyses, statistical normality tests were performed (Kolmogorov–Smirnov) to verify statistical test assumptions. Nonparametric tests were used for variables that did not confirm the normality assumptions, such as Kruskal–Wallis, chi-square, Wilcoxon, and Mann–Whitney. For the quartiles, when necessary, the Bonferroni test was used.

### 2.4. Ethical Aspects

This research was approved by the Research Ethics Committee of the Health School of the University of Brasilia, under No. 0372/2010, and followed the guidelines established by the Declaration of Helsinki. Each participant signed a free consent form agreeing to participate in the study. If necessary, researchers read the terms for the participant and if they did not feel comfortable to continue with the research, the next participant in line was invited to join the study.

## 3. Results

In total, 1232 individuals (57% male, *n* = 706) were evaluated in three days using 24 h recalls. The most frequent per capita income reported was between ½ to 1 minimum wage (Table 1). There were statistically significant differences between Brazilian’s income groups (*p* < 0.01). The North and Northeast region presented the lowest mean income in Brazil, being statistically different from the South (*p* < 0.01) and the Southeast (*p* < 0.01). The data resulting from the measurements for the customers revealed a population mostly above weight (overweight and obesity = 52.2%; *n* = 643) (Table 1).

Evaluating the female intake of sugar (sucrose), there is a statistical difference between the four different groups of sugars analyzed (*p* < 0.05). The differences occur between food prepared at home and sweetened drinks (*p* < 0.05), and between food prepared at home and added sucrose (0.005). For males, there was also a statistical difference among the groups of sucrose intake (*p* < 0.05), and only food prepared at home was similar to industrialized products (*p* = 1.00). There were no statistical differences among male and female for all the sugar (sucrose) groups (Table 1).

Total sucrose intake did not differ according to BMI (*p* = 0.321). However, the consumption of sugar (sucrose) for each classification group was different among the diverse types of sucrose intake (*p* < 0.01). For income, the only group without statistical difference among the types of sucrose intake was the no income group (*p* = 3.00). As for regions, only the Midwest presented a similar consumption of sucrose among the diverse sucrose intake groups (*p* = 2.00).

The differences between the five Brazilian regions for the intake of sucrose from industrialized products (*p* < 0.001), and products prepared at home (*p* = 0.005) were checked. For added sugar (sucrose) in liquids and sweetened drinks, there were no differences (*p* = 0.80 and *p* = 0.120, respectively).

Evaluating these groups of sugar (sucrose) intake and regions, the North presented the lowest intake from industrialized products and it was different from the Northeast (*p* = 0.007), the Southeast (*p* = 0.010), and the South (*p* = 0.043). Also, the north presented the lowest consumption for food prepared at home, being different from the Northeast (*p* < 0.001), the South (*p* < 0.001), and the Southeast (*p* < 0.001).

With the data obtained in this study, it was possible to calculate an average estimated energy requirement (EER) [29] of 2118.33 kcal ± 400.85. For individuals with low weight (BMI < 18.5 kg/m^2^), the EER was 1891.76 ± 388.76, and for normal weight (BMI: 18.5–24.9 kg/m^2^), the EER was 2157.05 ± 399.71. A significant difference in the EER between the two groups (*p* < 0.01 and F = 2.518) was verified. The mean of the total energy expenditure (TEE) obtained for the overweight (BMI: 25.0–29.9 kg/m^2^) was 2379.96 kcal ± 410.93 (*n* = 310), and for obese (BMI ≥ 30 kg/m^2^) 2498.36 kcal ± 468.22 (*n* = 167).

The TEV average consumption over 24 h was 1980.23 kcal ± 726.75, ranging from 369.23 to 5878.10 kcal for the three days of the week. The lowest TEV consumption occurred among females and represented individuals with only lunch consumption at the PR. The outliers of sucrose intake were removed to perform the analysis, and TEV was not considered for this removal. Such average TEV consumption represents a lower energy intake than needed for individuals, for both normal weights and those with overweight and obesity, corresponding to 91.3% of the normal weight needs.

There was no significant difference in sugar (sucrose) consumption among each of the three days (*p* = 0.078). When assessing the contribution of each item on the average of the three days, it was observed that the addition of sugar contributed to 36.7% of all sugar (sucrose) and sweetened beverages with 22.53%. Food prepared at home contributed 20.06% and industrialized products 22.53%.

On Sunday, sweetened drinks represented 30.06% of total sugar (sucrose) intake, while the addition of sugar consumption was constant throughout the week, representing 1/3 of the sugar ingested by the individuals. The average percentual contribution of sugar in the 24 h recall was 6.6%, with a minimum of 0% and a maximum of 59.2%.

Table 2 presents the sugar intake for each type of product during the weekend and during the weekdays.

## 4. Discussion

The average consumption per capita/day of soft drink, according to POF, was 94.7 g [3]. Using the average percentage of 10% of sugar (sucrose) in these drinks, this comes to 9.9 g of sugar, a very similar result to the average of 9.2 g found in this study. In Canada, a study showed that 663 g/day of regular soft drink was consumed [30], which means a very high consumption of sugar from this source, 66.6 g. It can be emphasized that because soft drinks have, on average, 10% sugar, a quantity of 100 mL represents a sugar intake above the current WHO recommendation and more than twice the target for this nutrient.

As for juices, powdered juices, and nectars, the Brazilian consumption according to POF was 146 g [3]. Using the average percentage of 12% of sugar in these drinks, this means a consumption per day of 17.5 g of sugar, a higher amount than the one found in this study (5.2 g). Brazilian low incomes were evaluated in the present study, and POF represents the consumption for the Brazilian population. It is important to mention that industrialized juices and nectars are more expensive than soft drinks in Brazil. In Canada, a study showed that the population had an mean intake of 572 g/day of sweetened juices [30], representing 68.6 g of sugar intake from this source of product, a very high consumption of sugar per day.

The North region presented the lowest income among the evaluated population of this study and probably explains the lowest sugar (sucrose) intake from industrialized products and sweetened drinks.

According to the UN food balance sheet data, the New Zealanders, on average, consume about 147 g/day (37 teaspoons) of sugar [31]. Souza et al. [32] showed that in 2002, 12.2 kg of granulated sugar and 6.1 kg of refined sugar per capita per year were consumed in Brazil. Considering a total of 18.3 kg and dividing this by 365 days corresponds to a consumption of 50.2 g of sugar per day [32], higher than the median values for the population of our study.

Marriott et al. [33] examined the intake of added sugars and selected nutrients from the 2003–2006 National Health and Nutrition Examination Survey data. Thirteen percent of the population had an added-sugar intake >25% of total energy [33].

In the present study, the average percentage is within the WHO recommendations to consume less than 10% of the total energy that comes from sugars [10]. However, in 2015, the WHO began to recommend that levels should be lower than 5% and, thus, this population would need to reduce their consumption [11], with the reduction being more important regarding the sugar (sucrose) added to consumed liquids and sweetened drinks.

A British study showed that ageing men (19–64 years) consume 15.2% (82 g/day) of non-milk extrinsic sugars (NMES) and women 13.5% (57.2 g/day). The NMES included all added sugars in processed and manufactured foods and drinks, and sugars in fresh fruit juices, honey, and syrups [34]. In the United States, a study showed that the intake of regular soft drinks and fruit drinks had a prevalence of 70%, where 23.9% of adults consumed these one or more times a day, and 10.7% two or more times a day [35].

In this study, the sugar (sucrose) intake from beverages on Sunday was higher than during the weekdays. Other studies found, similarly, that diet on weekends was less healthy than that on weekdays, and that diet quality on Saturday was the poorest. Compared with average weekday consumption, consumption on Saturday was associated with an increase in the daily intakes of total energy by 181.04 kcal (95% confidence interval = 141.98–220.10), and by 18.34 kcal (10.07–26.62) for the energy from sugar (sucrose)-sweetened beverages [36]. This finding corroborates with a Canadian study that found that the mean energy intake on weekends (2130 ± 18 kcal) was significantly higher than on weekdays by 62 ± 23 kcal, and that overall, dietary quality was significantly poorer on weekends than on weekdays [37]. There is limited data based on a high number of participants comparing the quality of consumption between the weekend and weekday.

It is necessary to change the high sugar (sucrose) intake worldwide, but this cannot be imposed by either politicians or the public. A consensus must be built to change this large sugar (sucrose) intake [38]. It is important to underline that, many times, there not been sufficient evidence for governments to take action [39]. For sugar-sweetened beverages, it has been suggested that energy in liquid form could be less satiating than when derived from solid foods, resulting in increased consumption [40].

As previously mentioned, in Brazil, the food culture is heavily influenced by the culture of sugar cane because of the immigration of several populations who brought a taste for cakes and sugary desserts, mainly from Portuguese colonization.

This study, however, has inferential limitations. Pregnant women and children were excluded from the sample due to their specific needs and methods for assessing nutritional status, or due to legal restrictions. Another limitation was the exclusion of diabetic customers, because this pathology restricts the consumption of sugars.

Data was collected using 24 h recall, and this method presents limitations because it depends on the participant’s memory and a well-trained interviewer to get detailed information, especially regarding sugar (sucrose) addition. A gold method would be a direct weighing of each item consumed. Another limitation of this study is the use of mean values to represent the sugar (sucrose) composition in industrialized products, food prepared at home, and sweetened drinks. Not all products present the same amount of sugar (sucrose), and there is variance among different available types.

## 5. Conclusions

Since this research study encompasses a population with low per capita income, the free sugar (sucrose) consumption is still the most significant contributor to the total consumption of sugar (sucrose), followed by sweetened drinks, especially during the weekend. This study was conducted using adults participating in a social program of great importance in Brazil. This social program uses simple and cheap ingredients for food preparation at lunchtime, the main meal consumed by Brazilians. Therefore, elaborated desserts using sugar (sucrose) in their preparation did not contribute to the sugar (sucrose) intake, since they are more expensive and more workers are required for their preparation. Also, these popular restaurants do not serve sweetened drinks at lunch as this would make the meals more expensive. These characteristics from PRs are positive for their customers, and do not encourage them to eat more sugary products.

Sugar intake of the participants did not follow the recommendations of WHO, of a maximum of 5% of TEV from sugar in the diet. Even though it was a low-income population, the sugar and sugary products consumed were above these limits. Since this population presents a high percentage of overweight and obese participants, new diseases associated with excessive sugar intake could easily appear.

Sweetened drinks are responsible for a high consumption of sugar (sucrose) during the weekends, but for weekdays, this low-income population adds free sugar (sucrose) to other kinds of drinks, leading to a pattern of high need of sugar (sucrose) content in other forms of consumption.

Evidently, in the Brazilian case, the progress of the social programs that drew thousands of people out of poverty must become associated with nutrition education programs and encouragement of a healthy lifestyle, seeking to prevent a situation whereby a change in social class could also be accompanied by the consumption of products rich in free sugars.

## Figures and Tables

**Table 1:** Frequency of participants by gender, income, nutritional status, and Brazilian regions, and a comparison of median sucrose consumption (g) by sugary product groups.

			Added Sugar (Sucrose)				Sweetened Drinks				Sweet Food Prepared at Home				Industrialized Food			
		*n*	Percentile 25 (g)	Median (g)	Percentile 75 (g)	*p* *	Percentile 25 (g)	Median (g)	Percentile 75 (g)	*p* *	Percentile 25 (g)	Median (g)	Percentile 75 (g)	*p* *	Percentile 25 (g)	Median (g)	Percentile 75 (g)	*p* *
Gender	female	526	5.00	10.24	17.50	0.723	0.00	4.50	10.00	0.326	0.00	0.00	10.00	0.554	0.00	2.00	10.00	0.122
	male	706	4.67	10.40	17.33		0.00	5.00	11.50		0.00	0.00	10.00		0.00	2.00	10.00	
Income	No income	56	3.33	7.73	16.75	0.571	0.00	0.00	7.00	0.155	0.00	0.00	12.00	0.291	0.00	0.00	8.00	0.135
	Up to ¼ of MW	109	5.00	8.65	16.67		0.00	4.50	9.50		0.00	0.00	7.00		0.00	0.00	7.00	
	¼ to ½ of MW	266	5.00	11.58	17.33		0.00	5.50	10.50		0.00	0.00	8.00		0.00	3.00	12.00	
	½ to 1 MW	472	5.23	10.33	17.83		0.00	5.50	12.00		0.00	0.00	9.00		0.00	2.00	10.00	
	>1 to 2 MW	245	4.67	10.11	17.00		0.00	4.50	10.00		0.00	0.00	10.00		0.00	1.00	10.00	
	>2 MW	76	5.58	10.92	18.94		0.00	6.00	10.75		0.00	0.00	12.00		0.00	2.00	12.00	
Nutritional status	Low-weight	60	5.25	10.92	20.00	0.781	0.00	5.75	9.00	0.948	0.00	0.00	13.00	0.418	0.00	0.00	10.00	0.721
	Normal weight	529	4.27	10.37	18.09		0.00	5.00	11.25		0.00	0.00	9.00		0.00	2.00	10.00	
	Overweight	454	5.10	10.24	16.92		0.00	5.00	10.50		0.00	0.00	10.00		0.00	1.00	10.00	
	Obesity	189	5.50	10.00	16.57		0.00	4.50	11.00		0.00	0.00	9.00		0.00	3.00	10.00	
Brazilian Region	North	168	10.17	17.40	0.00	0.80	3.50	9.50	0.00	0.120	0.00	6.00	0.00	0.005	0.00	3.00	6.25	<0.001
	Northeast	401	11.33	17.67	0.00		5.50	11.00	0.00		0.00	9.00	0.00		2.00	12.00	4.83	
	Midwest	39	6.67	10.67	2.50		6.00	15.00	0.00		0.00	10.00	0.00		0.00	7.00	3.50	
	Southeast	323	10.59	18.00	0.00		5.50	10.50	0.00		0.00	10.00	0.00		3.00	10.00	4.12	
	South	300	10.00	16.81	0.00		4.00	10.50	0.00		0.00	10.00d	0.00		3.00	10.00	4.95	
Total	Brazil	1232	4.83	10.25	17.33		0.00	5.00	10.50		0.00	0.00	10.00		0.00	2.00	10.00	

MW = minimum wage (US 244.02). BMI: Low-weight: <18.5 kg/m^2^; Normal weight: 18.5–24.9 kg/m^2^; Overweight: 25.0–29.9 kg/m^2^; Obesity: ≥30 kg/m^2^. * *p*-values represent comparisons among proportions (percentiles) within each variable (comparison of sugar intake between male and female, comparison of sugar intake among different incomes, comparison among different body mass index (BMI) classifications and comparisons among regions).

**Table 2:** Central tendency measure and variance of sugar (sucrose) consumption (g) of the low-income population in Brazil when comparing weekend and weekdays (*n* = 1232).

	Percentile 25 (g)	Median (g)	Percentile 75 (g)	*p* *
Added Sugar (sucrose) Weekend	0.00	10.00	18.00	0.81
Added Sugar (sucrose) Weekday	0.00	0.00	5.00	
Sweetened Drink Weekend	0.00	0.00	17.50	<0.001
Sweetened Drink Weekday	0.00	0.00	11.50	
Sweet Food Prepared at Home Weekend	0.00	0.00	0.00	0.20
Sweet Food Prepared at Home Weekday	0.00	0.00	3.00	
Industrialized Food Weekend	0.00	0.00	5.00	<0.001
Industrialized Food Weekday	0.00	0.00	9.00	

* *p*: Wilcoxon test.
